# Case report: “Congenital cutaneous langerhans cell histiocytosis presenting with blueberry Muffin Rash”

**DOI:** 10.3389/fped.2022.1073624

**Published:** 2022-12-22

**Authors:** Mariam Thalji, Asil Yagmour, Dania Alameh, Hanin Shatrit, Mais Inerat, Sami Issa Bannoura, Amir Atawneh, Motee Abuawaad

**Affiliations:** ^1^ Medical Research Club, Faculty of Medicine, Al-Quds University, Jerusalem, Palestine; ^2^ Departement of Pediatrics, Makassed Hospital, Jerusalem, Palestine; ^3^ Departement of Pathology, Makassed Hospital, Jerusalem, Palestine

**Keywords:** langerhans cell histiocytosis (LCH), muffin rash, hashimoto pritzker disease, pediatrics, hematology, dermatology, pediatric hematological diseases

## Abstract

Congenital cutaneous Langerhans cell histiocytosis-(LCH), named Hashimoto Pritzker disease, is a rare subtype among the clinical spectrum of LCH that often presents at birth or through the neonatal term and spontaneously resolve within a few months. In rare instances, infants with congenital cutaneous LCH may present with a blueberry-muffin rash. We reported a case of a male newborn who presented with blueberry muffin rash and was diagnosed with congenital cutaneous LCH later on. The diagnosis was confirmed by excluding other possible systemic causes of blueberry muffin rash, followed by a skin biopsy. Skin biopsy showed reticular dermis-hypodermis infiltration by medium-sized cells which had a pale eosinophilic cytoplasm and irregular nuclei. The lesional cells were positive for Langerin, CD1a, S100, and CD68 immunostains, consistent with congenital cutaneous LCH. Investigations were performed and revealed no systematic disease involvement. After a discussion with the pediatric Hemato-Oncologist, the decision was to keep track of a “wait-and-see” approach. Long-term follow-up revealed no recurrence of the cutaneous lesions or any systemic involvement, which further leads to congenital cutaneous LCH diagnosis. Even though it is very rare, blueberry muffin rash differential diagnosis should include congenital cutaneous LCH. Early recognition of this condition protects patients from unnecessary and possibly unsafe systemic treatment.

## Introduction

Langerhans cell Histiocytosis (LCH) is a rare condition described initially by Lichtenstein in 1953. It is defined by clonal proliferation and accretion of Langerhans cells in different tissues resulting in organ damage or malignancy formation. Although the mechanism behind this accumulation remains uncertain, LCH is supposed to have a neoplastic or inflammatory process (1–4). LCH mainly affects the pediatric population, with wide heterogeneity in clinical presentations and consequences. The incidence of LCH is about 5 per million children and cutaneous involvement presents in 40% of these cases (5).

Previously, LCH was classified into four subcategories: Letterer-Siwe disease, eosinophilic granuloma, Hand-Schüller-Christian disease, and Hashimoto-Pritzker disease. However, recently it has been specified as a clinical spectrum with a new classification based on involving one or more organ systems, one or more locations within a single organ, and involvement of high-risk organs, particularly the liver, spleen, and bone marrow (4–9). Congenital cutaneous LCH is generally a benign condition involving one-system, distinguished by generalized red-brown macules, papules, and nodules. The disease rarely involves the skeleton, lungs, eyes, and abdomen (10, 11).

The term “Blueberry muffin baby” (BMB) was initially used for the description of congenital rubella cutaneous manifestations in the 1960s (12). They are characterized by multiple, non-blanching, purple to dark blue macules, papules, or nodules. Rather than rubella, various diseases have been related to BMB, including congenital infections (e.g., Toxoplasmosis, Rubella, Cytomegalovirus, Herpes and other agents (TORCH), Parvovirus), hematologic disorders, metabolic disorders, neoplastic and other diseases (i.e., Langerhans cell histiocytosis, neonatal lupus). In rare cases, Congenital cutaneous LCH manifests clinically as BMB (13, 14). Herein, we describe an unusual Congenital cutaneous LCH case, who presented with blueberry muffin rash and had an excellent clinical outcome.

### Case presentation

**Figure 1:**
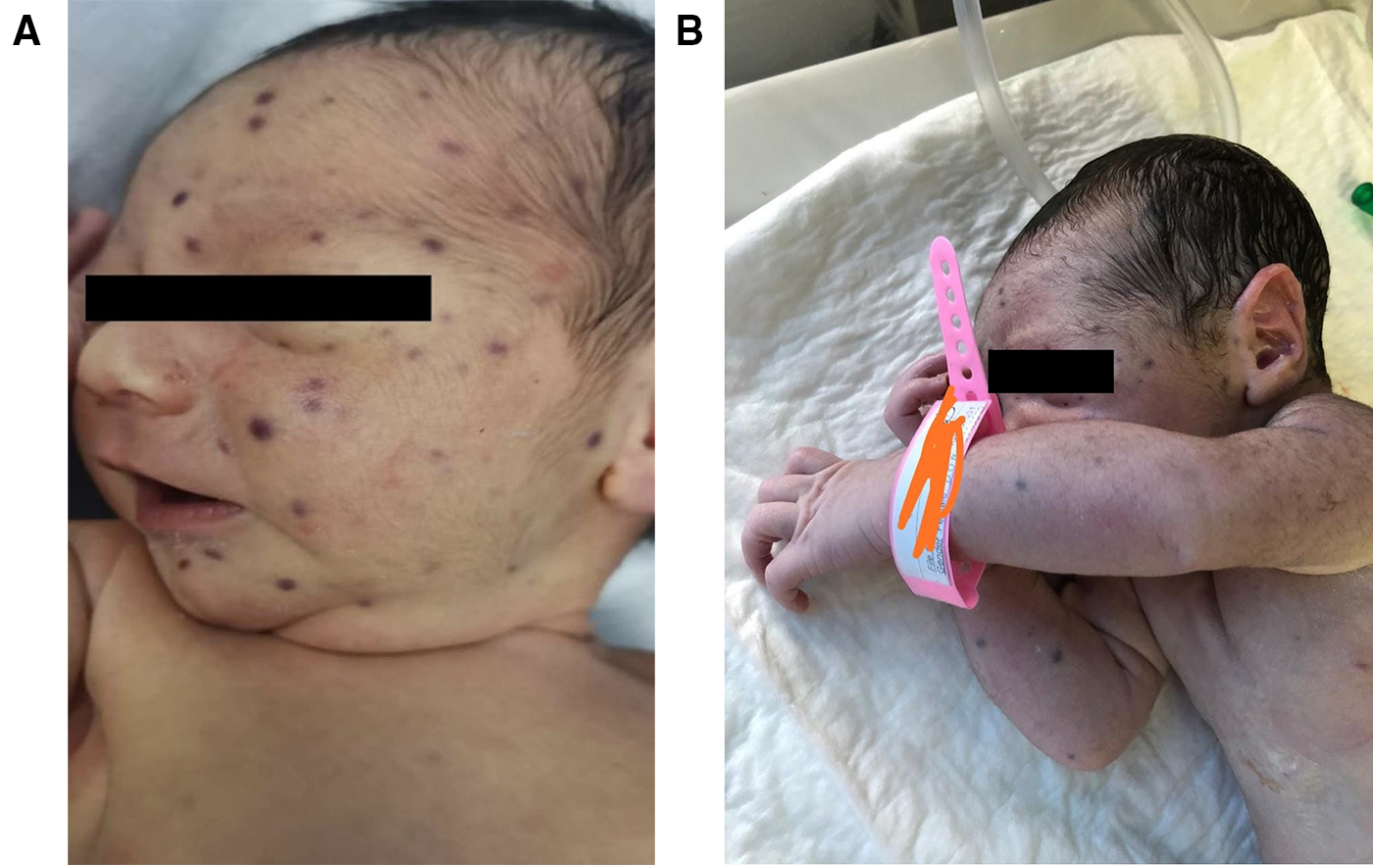
Blueberry muffin baby. (**A**) & (**B**) Disseminated dark red to purplish macules and papules on the face, neck, and upper limbs.

Our patient is a male newborn who was referred to our center at the age of 4 days with a blueberry muffin rash. He was born *via* normal vaginal delivery after an uneventful full-term pregnancy for a healthy 21-year-old primigravida woman. Physical examination revealed several scattered dark purple papules-like spots over the face, neck, and limbs of variable sizes (2–4 mm) (Figure 1). The rash was palpable with a smooth surface. There was no hepatosplenomegaly, jaundice, or ecchymosis. He had normal physical neuroexam. Transfontanelle ultrasound for brain was done and normal. Otherwise, the baby was active and healthy. No history of bruises, bleeding, fever, or abnormality in movement. His growth parameters were between the mean and −1 Standard Deviation (SD).

**Figure 2:**
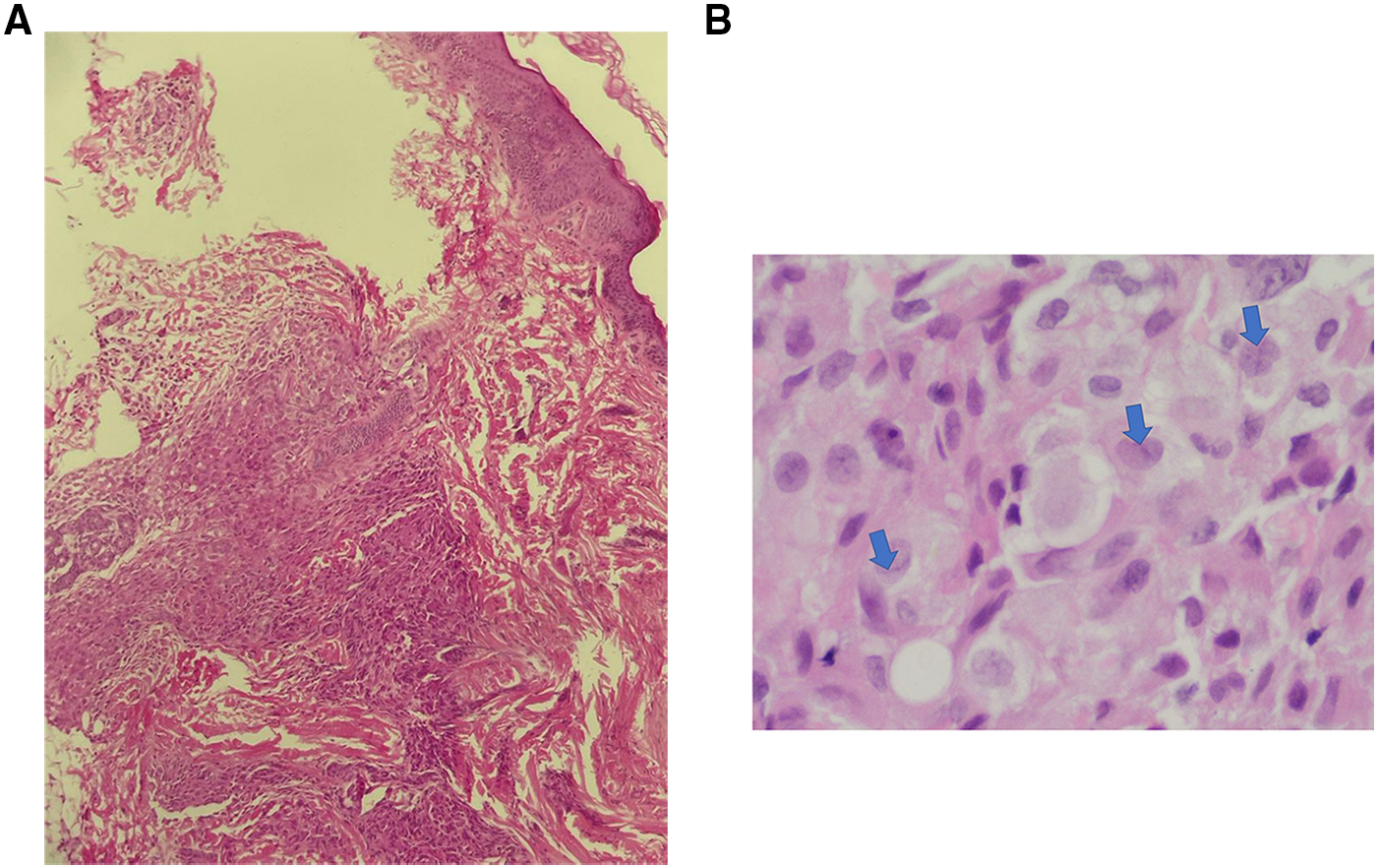
Congenital cutaneous langerhans cell histiocytosis. (**A**). Dermal- hypodermal infiltration by histocytes (H & E. 20X), (**B**). The lesional cells have abundant, light eosinophilic cytoplasm and irregular nuclei with frequent nuclear folds and grooves (arrows), obscure nucleoli and fine chromatin.

**Figure 3:**
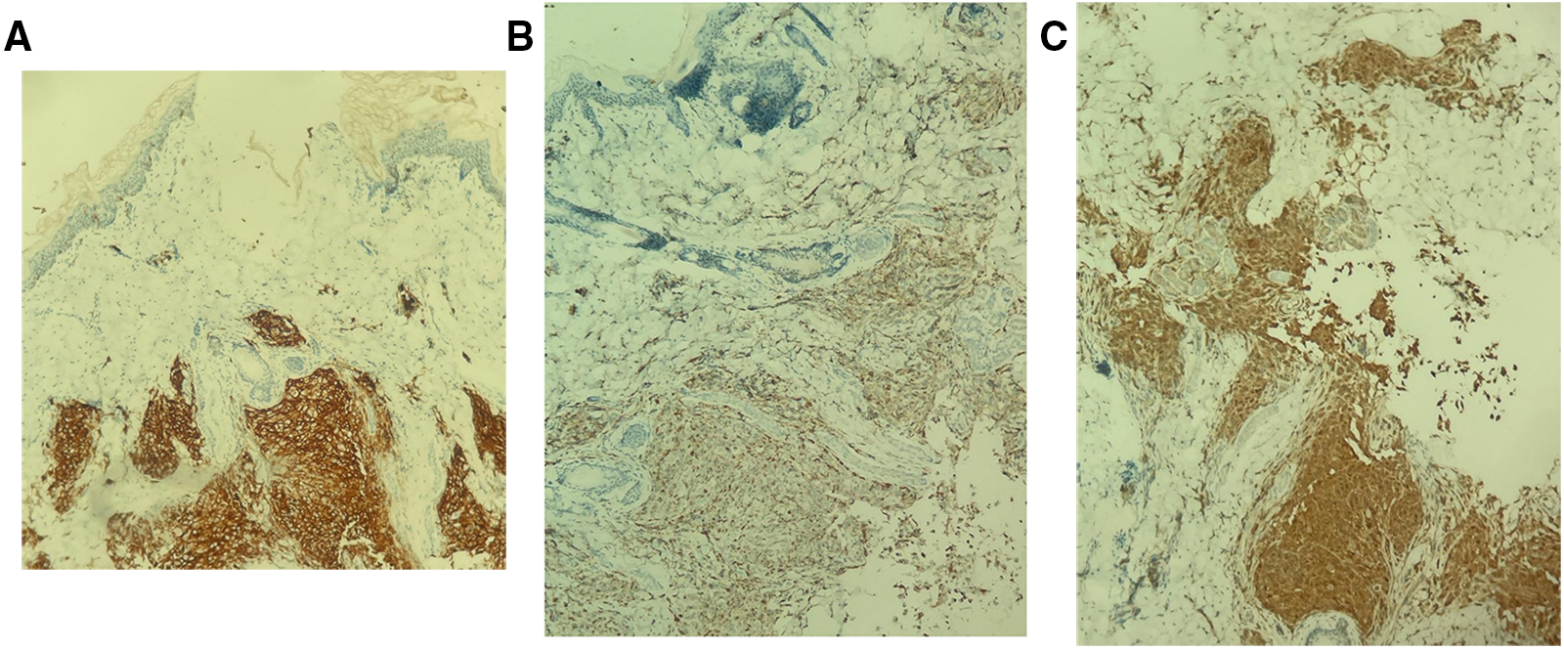
Immunohistochemistry. The lesional histocytes are diffusely positive for CD1a (**A**), CD68 (**B**), and S100 (**C**).

Multiple Investigations were performed, including complete blood count (CBC), blood serum chemistry, coagulation profile, liver function tests, and blood film, all of which were normal. Serology for congenital infections showed the following results; VDRL: non-reactive, TORCH: negative except for high cytomegalovirus (CMV) IgG antibody, and Qualitative PCR testing for urine and blood were negative. A urine sample analysis to rule out neuroblastoma did not show homovanillic acid (HVA) or Vanillylmandelic acid (VMA). Skin biopsy revealed a reticular dermis-hypodermis infiltration by medium-sized cells, which had a pale eosinophilic cytoplasm and irregular nuclei. The cells were positive for Langerin, CD1a, S100, and CD68 immunostains, consistent with cutaneous Langerhans cell histiocytosis (Figures 2,3). Further investigations were performed to exclude systemic involvement, including a skeletal survey with a skull view, and abdominal ultrasound, all of which were normal, and supported the diagnosis of congenital cutaneous LCH.

After a discussion with the pediatric Hemato-Oncologist, a “wait-and-see” approach was preferred. The infant remained stable and active during his stay with good general condition. Labs were followed up and showed good results; the rash faded with time. He was discharged home with recommendations to follow up with a pediatric hemato-oncologist. At the age of two months, almost the whole lesions on his skin have resolved. His last follow up was at the age of two years, there was no evidence of recurrence or any extracutaneous manifestations. No continuity of follow up beyond this age.

### Discussion

Blueberry muffin baby (BMB) is a clinical manifestation caused by extramedullary hematopoiesis within the dermis. Clinically, it is characterized by diffuse dark bluish to purple papules or nodules. BMB appearance had a broad spectrum of differential diagnoses, including congenital infections (TORCH and Epstein Barr virus), malignancies/proliferative conditions (neuroblastoma, congenital leukemia, congenital rhabdomyosarcoma, LCH), hematologic disorders (rhesus hemolytic anemia, hereditary spherocytosis, ABO blood groups incompatibility, and twin-twin transfusion syndrome) (14–16). Congenital Cutaneous LCH is an infrequent presentation among the BMB differentials (17).

Congenital cutaneous LCH is considered a rare variant of single-site LCH, first reported by Hashimoto and Pritzker in 1973 (10, 11). Most cases are present at birth or later in the neonatal term with cutaneous manifestations represented by reddish-brown or violaceous papules or nodular lesions. The disease clinical course is often benign with a spontaneous –resolution tendency. However, in a few patients, the disease may progress to multi-systemic LCH affecting other organs, especially the spleen, liver, bone, and lymph node (11, 15, 16, 18).

Besides the disease's clinical course, skin biopsy demonstrates histologic and immunohistochemical findings are crucial in the diagnosis. The typical histopathological appearance of cutaneous LCH shows infiltration of the dermis by cells that have abundant, light eosinophilic cytoplasm and irregular nuclei with frequent nuclear folds and grooves, obscure nucleoli, and fine chromatin. Some inflammatory cells, such as lymphocytes, eosinophils, neutrophils, and mast cells, may coexist. Immunohistochemical stains, including S100, CD207 (langerin), and CD1a, are essential to confirm the diagnosis. The gold standard is evidence of Birbeck granules on electron microscopy (15, 16, 18).

Regarding our case, a step-wise plan was followed to investigate the cause of the patient's BMB presentation. Congenital infection was our first concern; therefore, extensive infectious workup was done, which revealed negative results except for high levels of CMV IgG anti-body. Hematological malignancies were unlikely, with a normal CBC and blood film. Also, neuroblastoma was excluded by a normal urine level of VMA and HMA. A skin biopsy confirmed the diagnosis of LCH. To look for other body systems involvement, a skeletal survey was done, including skull views as it is the most common site for LCH and it was free of any lesions. Additionally, the abdominal ultrasound was normal. The rash gradually faded and it disappeared entirely by the age of two months. His last follow-up at the age of two years has revealed no evidence of recurrence or any extracutaneous manifestations.

A comprehensive literature review on PubMed, Google Scholar, and Embase revealed 11 cases of congenital cutaneous LCH present with BMB, including the current case. All cases had spontaneous resolution of the disease during the first few weeks of life, ranging from 15 days up to 18 months without requiring any treatment. In our case, the rash disappeared at the age of two months. Follow up period in the reported cases ranging from 11 months till 2 years and none of those cases showed evidence of recurrence (2, 17, 19–22).

Since most cases of isolated cutaneous LCH has a self-limited course within a few months, the treatment is usually conservative. A careful watchful waiting approach is approved in many cases which is comparable to our case. Topical steroids may also be used in non-resolving cases. Furthermore, severe symptomatic cutaneous lesions necessitate the use of other treatment options such as topical nitrogen mustard or psoralen with ultraviolet light therapy (17, 18). Close monitoring for any signs of recurrence or progression to multisystem disease is crucial. Following the early thorough workup, all newborns should be monitored by a careful clinical examination, CBC, and possibly ultrasounds. A potentially serious multisystem disease progression might happen within several weeks to months, for which systemic therapy is indicated (16).

Our case has some limitations, lack of follow up information at time of writing the report, and parents didn't adhere to follow up in our hospital.

## Conclusion

To summarize, our case highlights the importance of raising the index of suspicion toward considering congenital cutaneous LCH as a cause of BMB. A comprehensive diagnostic approach should be implemented to exclude infectious and systemic causes as well as skin biopsy findings, which are critical for the diagnosis and thus allow the wait-and-observe approach. Regular follow-up is mandatory in these patients for the detection of any possible relapses or disease progression.

## Data Availability

The original contributions presented in the study are included in the article/Supplementary Material, further inquiries can be directed to the corresponding author/s.
